# Hair Barrette Induced Cochlear Implant Receiver Stimulator Site Infection with Extrusion

**DOI:** 10.1155/2015/510741

**Published:** 2015-01-05

**Authors:** Trung N. Le, Jordan Hochman, Darren Leitao

**Affiliations:** Department of Otolaryngology Head and Neck Surgery, University of Manitoba, Health Sciences Centre, GB421-820 Sherbrook Street, Winnipeg, MB, Canada R3E 0V9

## Abstract

*Background*. Cochlear implant infections and extrusion are uncommon but potentially devastating complications. Recent literature suggests conservative management can be employed. Local measures inclusive of aggressive surgical debridement with vascularized flaps and parenteral antibiotics represent a viable option and often permit device salvage. However, explantation should be considered if there is evidence of systemic, intracranial, or intractable infection. *Method*. A Case report and literature review. *Case Report*. This case illustrates a complicated local wound infection associated with cochlear implantation due to transcutaneous adherence of a ferrous hair barrette to a cochlear implant magnet. Reconstruction of computed tomography (CT) data with 3D volume rendering significantly improved the value of the images and facilitated patient counseling as well as operative planning. *Conclusion*. Cochlear implant infections can be associated with foreign bodies. CT images are beneficial in the evaluation of cochlear implant complications. 3D CT images provide a comprehensive view of the site of interest, displaying the relationship of the hardware to the skull and soft tissues, while minimizing associated artifacts. Cochlear implant patients should consider use of nonmetallic hair devices.

## 1. Case Presentation

**Figure 1:**
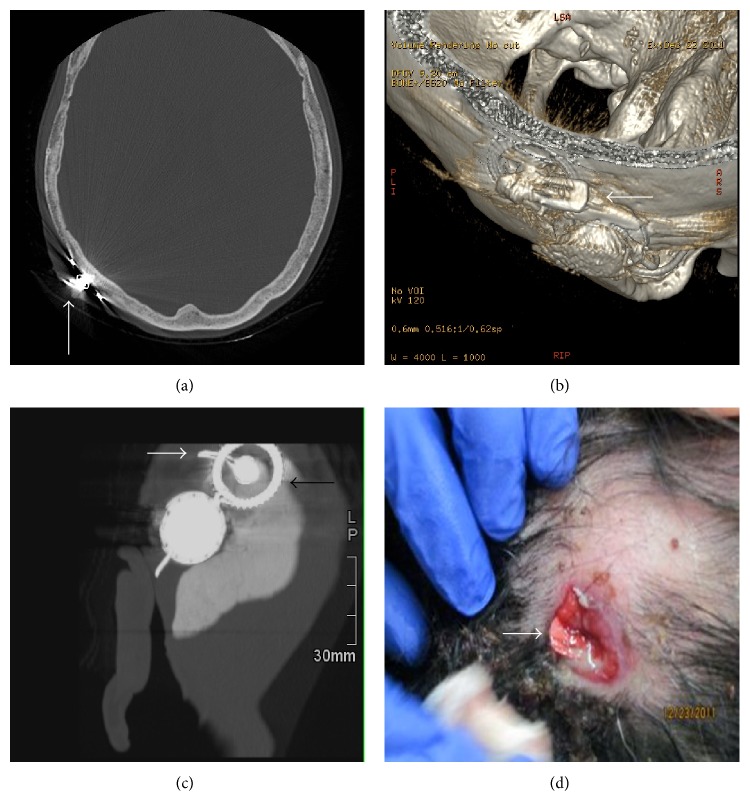
CT images ((a) axial view, (b) multiplanar reconstruction with maximum-intensity projection, and (c) 3D volume rendering) and (d) intraoperative findings of the barrette hair clip (white arrows) and its spatial relationship with the magnet of the receiver stimulator (black arrow).

A 56-year-old woman presented with infection in the right parietal skull. She had previously undergone a right-sided cochlear implant in a remote implant centre 9 years before. The patient's* de novo* presentation to our facility was secondary to a one-month history of tenderness and swelling at the surgical site. Her device was still in use. Inspection found a mat of congealed hair and purulence immediately overlying the receiver stimulator. Gentle manipulation suggested an exposed device. The patient underwent a noncontrast CT scan of temporal bone with 0.625 mm axial cuts (Figure 1(a)) to look for extent of infection within the mastoid and temporal bone, as well as meningitis. Artifact was demonstrated, but we confirmed no extent of infection to mastoid or brain. She was placed on intravenous antibiotics and taken for an urgent operative debridement.

At surgery, debridement of the wound revealed that the metallic structure visible was not the cochlear implant; rather, it was a metallic hair barrette that was stuck to the magnet of the receiver stimulator (Figure 1(d)). The wound was pulse-irrigated with bacitracin. All surrounding soft tissues were debrided. The receiver stimulator well was freshened with an otic burr. The device was maintained* in situ*. Vascularized local flap measures were undertaken. Unfortunately, due to later complication with meningitis, the implant device was extracted and the patient continued with parenteral and subsequently oral antibiotics. The patient is currently well with no long-term sequelae and is to have cochlear implant surgery on the contralateral side.

## 2. Discussion

Severe sensorineural hearing loss is a devastating disability. Cochlear implantation permits the deaf to access voiced sound and environmental noise. Infection and extrusion are uncommon but potentially devastating complications when they occur [1]. Improvements in surgical incisions, local wound care measures, and antibiotic prophylaxis have reduced the frequency of either a spontaneous or self-generated process [1–3].

Recent literature suggests conservative management can be employed, inclusive of aggressive surgical debridement and vascularized flaps in association with adjunctive parenteral antibiotics. Such local measures represent a viable option and often permit device salvage [2, 3]. However, explantation should be considered if there is evidence of systemic, intracranial, or intractable infection [2, 3].

Computed tomography scans are an essential part of the evaluation of temporal bone infections and cochlear implant complications [1]. One limitation of CT scans is the beam-hardening artifact caused by metallic implants, such as cochlear implant, metallic foreign body, and other ossicular prostheses [4]. Metals, which have high Hounsfield unit values (usually 1,000–4,000 HU), result in the attenuation of X-rays, producing gaps in CT projection data and significant starburst artifacts or streaking (Figure 1(a)) [4]. With the optimization of CT parameters and the evolution of 3D CT, metal-related artifacts can be diminished, allowing diagnostic examinations in most clinical scenarios [5]. We have found that, after the axial acquisition and reconstruction of the data sets, multiplanar (MPR) (Figure 1(b)) and 3D volume rendering (Figure 1(c)) CT views give significant benefit in providing clear details and information to aid in management. Retrospectively, we suspect an X-ray of the site could have been a low-cost screen for the presence of a foreign body.

This case is an illustration of a complicated local wound infection associated with cochlear implantation. 3D CT images provide a comprehensive view of the site of interest, displaying the relationship of the hardware to the skull and soft tissues, while drastically minimizing associated artifacts, a decided advantage over traditional axial and even MPR views.
